# Androgen receptor signaling at the tumor-immune interface in castration-resistant prostate cancer: single-cell transcriptomic analysis with AR-V7-related RT-qPCR context

**DOI:** 10.3389/fgene.2026.1945967

**Published:** 2026-10-27

**Authors:** Chuanmin Guo, Lejun Wu, Lintang Xu

**Affiliations:** Department of Urology, The First People’s Hospital of Lin’an District, Hangzhou, Zhejiang, China

**Keywords:** androgen receptor, androgen receptor splice-variant 7, castration-resistant prostate cancer, real time quantitative polymerase chain reaction, single-cell RNA-seq, tumor microenvironment

## Abstract

**Background:**

Androgen receptor splice-variant 7 (AR-V7) is associated with treatment resistance in castration-resistant prostate cancer (CRPC), whereas its relationship with the tumor-immune microenvironment remains incompletely defined.

**Methods:**

We performed secondary scRNA-seq analysis of the public CRPC dataset GSE137829 using Seurat-based preprocessing, dimensionality reduction, cell annotation, trajectory inference, SCENIC analysis, pathway enrichment analysis, and CellPhoneDB analysis. Because the public dataset does not provide validated AR-V7 status at the patient or single-cell level, it was not used for AR-V7-positive versus AR-V7-negative classification. RT-qPCR in 22Rv1 and LNCaP cells was retained as supportive analysis of selected AR-associated genes.

**Results:**

The single-cell dataset demonstrated substantial epithelial, immune, and stromal heterogeneity in CRPC and supported analysis of AR-associated transcriptional states and candidate regulatory programs. RT-qPCR showed higher KLK3, STEAP2, HOXB13, and NKX3-1 expression in 22Rv1 than in LNCaP cells; these differences are interpreted as supportive AR-associated observations rather than AR-V7-specific mechanistic validation.

**Conclusion:**

The revised study provides an exploratory transcriptomic characterization of AR-associated cellular states in CRPC. The findings are hypothesis-generating and do not establish AR-V7-specific causality or clinical prediction. Functional perturbation studies and datasets with directly measured AR-V7 status are required.

## 1 Introduction

Prostate cancer is one of the most frequently diagnosed malignancies in men and remains a major cause of cancer mortality worldwide (Sung et al., 2021). Progression to castration-resistant prostate cancer (CRPC) is characterized by continued tumor growth despite castrate androgen levels and is sustained in many tumors by persistent androgen receptor (AR) signaling, AR amplification, altered co-regulator activity, lineage plasticity, and expression of constitutively active AR splice variants. Among these variants, AR-V7 has received particular attention because it lacks the ligand-binding domain while retaining transcriptional activity. Clinical studies have shown that detection of AR-V7 in circulating tumor cells is associated with resistance to abiraterone and enzalutamide and with adverse outcomes in advanced CRPC (Antonarakis et al., 2014; Armstrong et al., 2019).

AR-V7 biology extends beyond a simple marker of resistance to ligand-directed AR inhibition. Molecular profiling of CRPC has shown that AR-V7 expression emerges during castration resistance and is embedded within broader transcriptional reprogramming of prostate lineage, cell cycle, stress response, and survival pathways (Sharp et al., 2019). In parallel, preclinical work has linked androgen pathway resistance states with altered immune-checkpoint expression; for example, PD-L1 upregulation has been reported in enzalutamide-resistant prostate cancer models (Bishop et al., 2015). These observations provide a rationale for examining whether AR/AR-V7-related transcriptional states coexist with immune-regulatory programs. Nevertheless, such coexistence should be interpreted as association unless AR-V7 is directly perturbed in an isogenic experimental system.

PSMA-targeted radioligand therapy provides an important therapeutic context for advanced CRPC. In the randomized phase II TheraP trial, 177Lu-PSMA-617 produced a higher prostate-specific antigen (PSA) response rate than cabazitaxel in selected patients with metastatic CRPC (Hofman et al., 2021). The phase III VISION trial subsequently demonstrated improvements in radiographic progression-free survival and overall survival when 177Lu-PSMA-617 was added to standard care (Sartor et al., 2021). Radiation can also influence antitumor immunity through immunogenic cell-death-associated processes and tumor antigen release, providing a biological basis for investigating interactions between tumor cell state and immune signaling (Galluzzi et al., 2020). In the present revision, however, the retrospective clinical RLT cohort and its regression, dosimetry, IHC, flow cytometry, and composite score analyses have been removed; RLT is therefore discussed only as translational context rather than as an outcome tested in the current analysis.

Single-cell RNA sequencing (scRNA-seq) is well suited to resolving epithelial, immune, and stromal heterogeneity in CRPC. We therefore reanalyzed the publicly available GSE137829 dataset, originally generated from CRPC biopsies to investigate lineage-state heterogeneity and luminal neuroendocrine transdifferentiation (Dong et al., 2020). The current study uses this dataset to characterize cellular composition, AR-associated transcriptional programs, differential expression, pathway enrichment, pseudotime dynamics, regulatory/co-expression structure, and inferred cell–cell communication. Importantly, GSE137829 does not provide a clinically validated AR-V7-positive/negative label for each cell. Consequently, AR-V7-related high/low groups and scores shown in the bioinformatics analyses are treated as computational transcriptomic signatures rather than direct measurements of AR-V7 status.

The objective of the revised study is to define AR-associated and AR-V7-related transcriptomic features within the CRPC single-cell landscape and to place these features in the context of tumor cell state and immune communication. RT-qPCR in 22Rv1 and LNCaP cells is retained as supportive analysis of selected AR/prostate lineage genes, but not as proof of an AR-V7-specific immune evasion mechanism. This narrower design preserves the bioinformatics analyses while avoiding causal or clinical predictive claims that are not supported by the available data.

## 2 Materials and methods

### 2.1 Data source and study design

The study was designed as a secondary bioinformatics analysis of publicly available scRNA-seq data, supplemented by RT-qPCR observations in prostate cancer cell lines. Single-cell data were obtained from the NCBI Gene Expression Omnibus (GEO) under accession GSE137829. The source dataset comprises CRPC biopsy-derived single-cell transcriptomes from six patients and was originally reported by Dong et al. (2020). No retrospective clinical treatment response cohort is analyzed in the revised manuscript. The GEO metadata do not provide validated patient-level or cell-level AR-V7 positivity; therefore, computational AR-V7-related scores are explicitly distinguished from direct clinical AR-V7 measurements.

### 2.2 Single-cell quality control

Cell-level quality assessment considered the number of detected genes, total unique molecular identifier (UMI) counts, and the proportion of mitochondrial transcripts. To avoid the overly restrictive 2,000–5,000-gene filter used in the earlier draft, the revised workflow follows the broader quality control framework reported for GSE137829, retaining cells with approximately 500–7,000 detected genes and mitochondrial transcript fraction below 10%. Cells outside these ranges were considered potentially low-quality cells or multiplets and were excluded. Quality control distributions were inspected before downstream analysis. General principles for single-cell quality assessment and exclusion of low-quality cells followed established scRNA-seq practice (Stuart et al., 2019; Hao et al., 2021; Luecken and Theis, 2019; Ilicic et al., 2016).

### 2.3 Normalization, feature selection, and dimensionality reduction

Downstream preprocessing was performed using a Seurat-based workflow (Stuart et al., 2019; Hao et al., 2021). Expression matrices were normalized to reduce library size effects, followed by identification of highly variable genes for dimensionality reduction. Data were scaled before principal component analysis (PCA). PCA was used to summarize major axes of transcriptional variation, and informative components were carried forward for nonlinear embedding. Uniform Manifold Approximation and Projection (UMAP) and t-distributed stochastic neighbor embedding (t-SNE) were used to visualize transcriptionally related cell states in two dimensions; UMAP was additionally displayed in three dimensions for descriptive visualization (Becht et al., 2019).

### 2.4 Cell clustering and cell type annotation

Cells were grouped according to transcriptomic similarity and visualized as discrete clusters. Cluster identity was assigned using canonical lineage markers together with reference-informed interpretation. Major compartments included epithelial, luminal, basal, endothelial, fibroblast, T-cell, macrophage, and natural killer (NK)-cell populations. Marker selection was guided by established reference-based annotation approaches and curated cell marker resources (Aran et al., 2019; Zhang et al., 2019). Expression patterns were evaluated across clusters and, where available, across individual source patients to assess inter-cluster and inter-patient heterogeneity.

### 2.5 AR/AR-V7-related transcriptomic signature analysis

AR pathway and prostate lineage genes, including *AR*, *KLK3*, *TMPRSS2*, *NKX3*-*1*, *FKBP5*, *KLK2*, *ACPP*, and *FOLH1*, were examined across clusters to characterize variation in androgen-related transcriptional activity. The bioinformatics workflow also generated an AR-V7-related transcriptomic signature score and a computational high/low grouping. Because direct AR-V7 splice-variant measurements are unavailable in GSE137829, this score was treated as an exploratory expression signature. It was not considered equivalent to CTC-based AR-V7 positivity, and all downstream comparisons using the score were interpreted at the signature level.

### 2.6 Differential expression analysis

Differentially expressed genes were identified between defined transcriptomic states or computational signature groups. Effect direction and magnitude were summarized using log2 fold change, and statistical significance was evaluated with multiple-testing correction. Differential expression results were displayed using ranked gene plots, clustered heatmaps, MA plots, volcano plots, and circular summaries. Interpretation emphasized robust expression patterns rather than isolated genes, consistent with recognized limitations of differential expression testing in single-cell datasets (Soneson and Robinson, 2018).

### 2.7 Functional enrichment analysis

Genes prioritized from differential expression or co-expression analyses were subjected to Gene Ontology (GO) and Kyoto Encyclopedia of Genes and Genomes (KEGG) enrichment analysis. Enriched terms were organized by biological theme and visualized using bubble plots, treemaps, sunburst diagrams, term networks, and gene concept networks. Functional enrichment was used to identify overrepresented biological processes and signaling pathways rather than to infer pathway activation causally. Enrichment analysis and visualization followed the general framework implemented in clusterProfiler (Yu et al., 2012).

### 2.8 Pseudotime and trajectory analysis

To explore continuous transcriptional transitions, cells were ordered along an inferred pseudotime axis. Trajectory reconstruction was used to visualize relationships among transcriptional states and to examine dynamic changes in selected AR/prostate lineage genes across the inferred continuum. Graph-based trajectory concepts were interpreted in accordance with established pseudotime and partition-based graph abstraction approaches (Trapnell et al., 2014; Wolf et al., 2019). Because the dataset is cross-sectional, pseudotime represents a computational ordering of cell states and not longitudinal follow-up of individual tumor cells.

### 2.9 Gene regulatory and co-expression network analysis

Gene regulatory and co-expression relationships were explored to identify coordinated transcriptional programs. SCENIC-based concepts were used to evaluate transcription-factor-centered regulon structure and cell-state-associated regulatory activity (Aibar et al., 2017). Co-expression networks were additionally constructed for AR/AR-V7-related genes to summarize positive and negative expression relationships. Network edges were interpreted as statistical or regulatory hypotheses and not as evidence of direct physical interaction or causal transcriptional control.

### 2.10 Cell–cell communication analysis

Intercellular communication was inferred from coordinated ligand and receptor expression across annotated cell populations. CellPhoneDB-based ligand–receptor analysis was used to summarize candidate interactions among epithelial, luminal/basal, fibroblast, endothelial, T-cell, macrophage, and NK cell compartments (Efremova et al., 2020). Interaction strength and direction were represented using ligand–receptor matrices, sender–receiver diagrams, interaction networks, circle plots, chord diagrams, and pathway-level communication summaries. These analyses identify putative communication programs and require experimental validation before biological causality can be assigned.

### 2.11 Signature-based ROC, survival, and correlation analyses

Within the bioinformatics component, the computational AR-V7-related signature was evaluated using receiver operating characteristic (ROC) analysis together with comparator expression/signature measures. Samples or annotated observations were also divided into computational signature high and signature-low groups for exploratory Kaplan–Meier analysis where survival annotation was available in the analysis table. Correlation analysis was used to examine relationships between the signature and available annotation variables, including PSA level, Gleason score, age, tumor stage, Ki-67 index, AR expression, and metastasis. These analyses are distinct from the deleted 78-patient retrospective clinical regression study and are not presented as a validated clinical prediction model.

### 2.12 Data visualization

Bioinformatics results were summarized using complementary visualization strategies, including PCA biplots, ridge plots, UpSet plots, dot plots, violin plots, heatmaps, UMAP/t-SNE embeddings, density contours, Sankey/river diagrams, network graphs, circular plots, chord diagrams, volcano plots, MA plots, and trajectory heatmaps. Visualization was used to display cluster structure, expression distributions, enrichment patterns, and inferred communication in a form that allowed cross-method comparison.

### 2.13 RT-qPCR analysis

RT-qPCR was performed for KLK3, STEAP2, HOXB13, and NKX3-1 in 22Rv1 and LNCaP prostate cancer cells. Total RNA was extracted, reverse-transcribed to complementary DNA (cDNA), and amplified using SYBR Green chemistry. GAPDH served as the internal reference gene, and relative expression was calculated using the 2^−ΔΔCt^ method. Experiments were performed in triplicate. Because 22Rv1 and LNCaP are non-isogenic cell lines with multiple genomic and transcriptional differences, the comparison was used only as supportive evidence for selected AR/prostate lineage transcriptional features and not as functional validation of AR-V7-mediated immune suppression.

### 2.14 Statistical analysis

All statistical tests were two-sided unless otherwise specified. For transcriptomic analyses, multiple comparisons were controlled using false-discovery-rate-adjusted significance where applicable. RT-qPCR results were summarized from triplicate experiments and compared between cell lines, with *p* < 0.05 considered statistically significant. The revised manuscript contains no multivariable Cox model, propensity score model, bootstrap-derived clinical ROC model, dosimetry–outcome regression, or composite Immune Evasion Risk score from the deleted retrospective clinical cohort.

## 3 Results

### 3.1 scRNA-seq quality control and cellular heterogeneity

Quality control visualization showed broad distributions of detected genes, UMI counts, and mitochondrial transcript fractions across cells (Figure 1F; Figure 5G). PCA-based summaries demonstrated that transcriptional variance was distributed across multiple components, and the PCA biplot highlighted genes contributing strongly to the principal axes (Figures 1B, 3C). UMAP and t-SNE embeddings resolved multiple transcriptionally distinct clusters (Figures 4D–F). These plots support substantial cellular heterogeneity in the public CRPC dataset; they do not by themselves establish AR-V7-defined biological groups.

**FIGURE 1:**
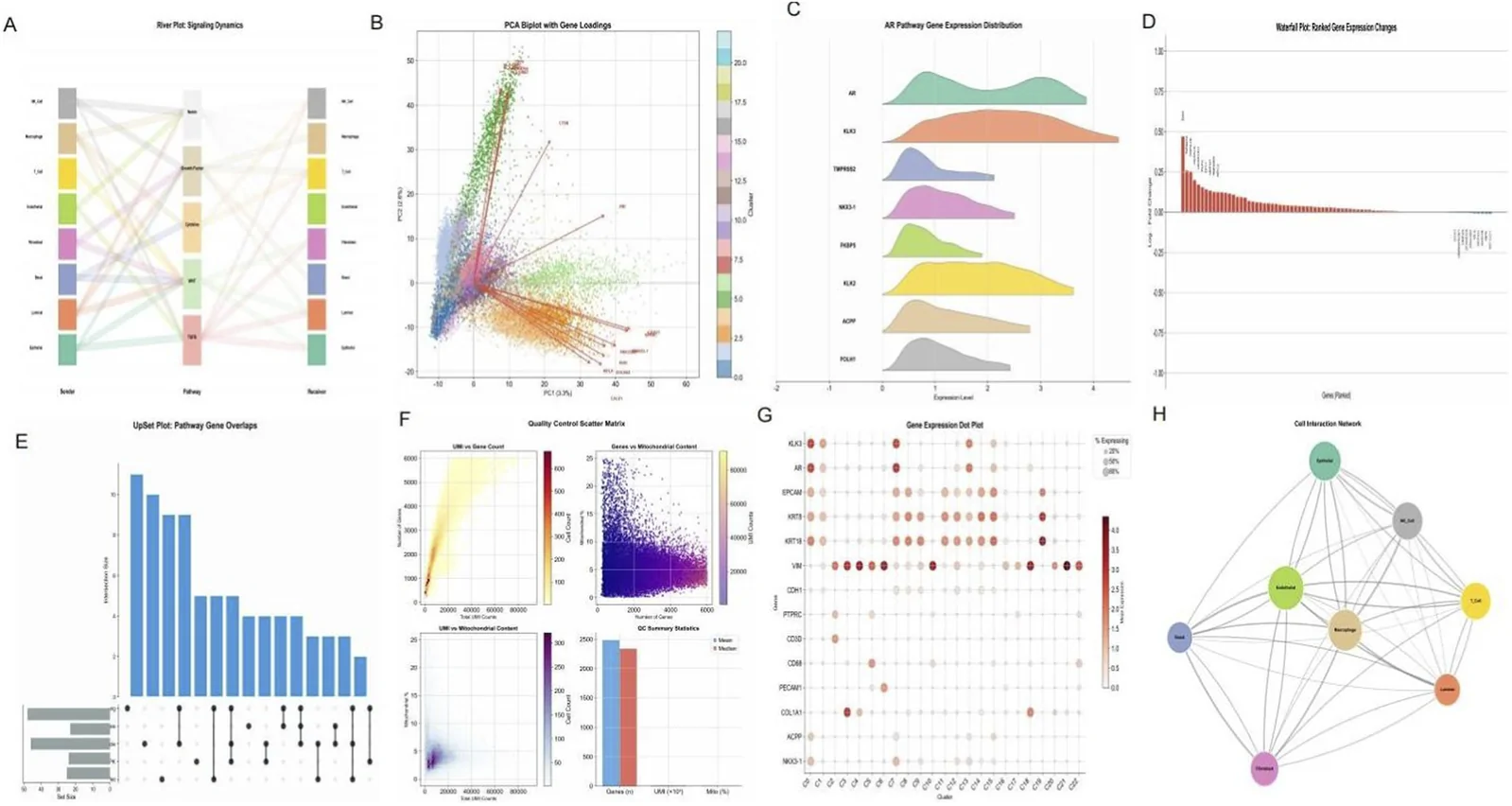
Overview of signaling, dimensionality reduction, AR pathway expression, quality control, marker expression, and cell interaction structure. **(A)** River plot summarizing inferred sender–pathway–receiver signaling dynamics among major cell types. **(B)** PCA biplot showing cell distribution and representative gene loadings. **(C)** Ridge plots showing expression distributions of selected AR/prostate lineage genes (*AR*, *KLK3*, *TMPRSS2*, *NKX3-1*, *FKBP5*, *KLK2*, *ACPP*, and *FOLH1*). **(D)** Waterfall plot of ranked gene expression changes. **(E)** UpSet plot showing overlap among pathway/gene sets. **(F)** Quality control scatter matrix displaying UMI gene count relationships, gene/mitochondrial content, UMI/mitochondrial content, and summary QC statistics. **(G)** Dot plot of canonical epithelial, immune, endothelial, and stromal marker expression across clusters. **(H)** Cell interaction network among major annotated cell types. Interaction edges represent computationally inferred communication and not experimentally validated signaling.

### 3.2 Cell population landscape and CRPC cellular heterogeneity

Canonical marker visualizations supported annotation of epithelial/luminal/basal, endothelial, fibroblast, T-cell, macrophage, and NK cell populations. The gene expression dot plot and stacked violin plot demonstrated cluster-restricted expression of epithelial markers (including EPCAM and keratins), mesenchymal/stromal markers (including VIM and COL1A1), and immune markers such as CD3D, CD68, and CDH1-related epithelial features (Figures 1G, 2G). Marker expression summaries across clusters and patients further illustrated inter-cluster and inter-patient heterogeneity (Figures 3A, 4C,G, 6G).

**FIGURE 2:**
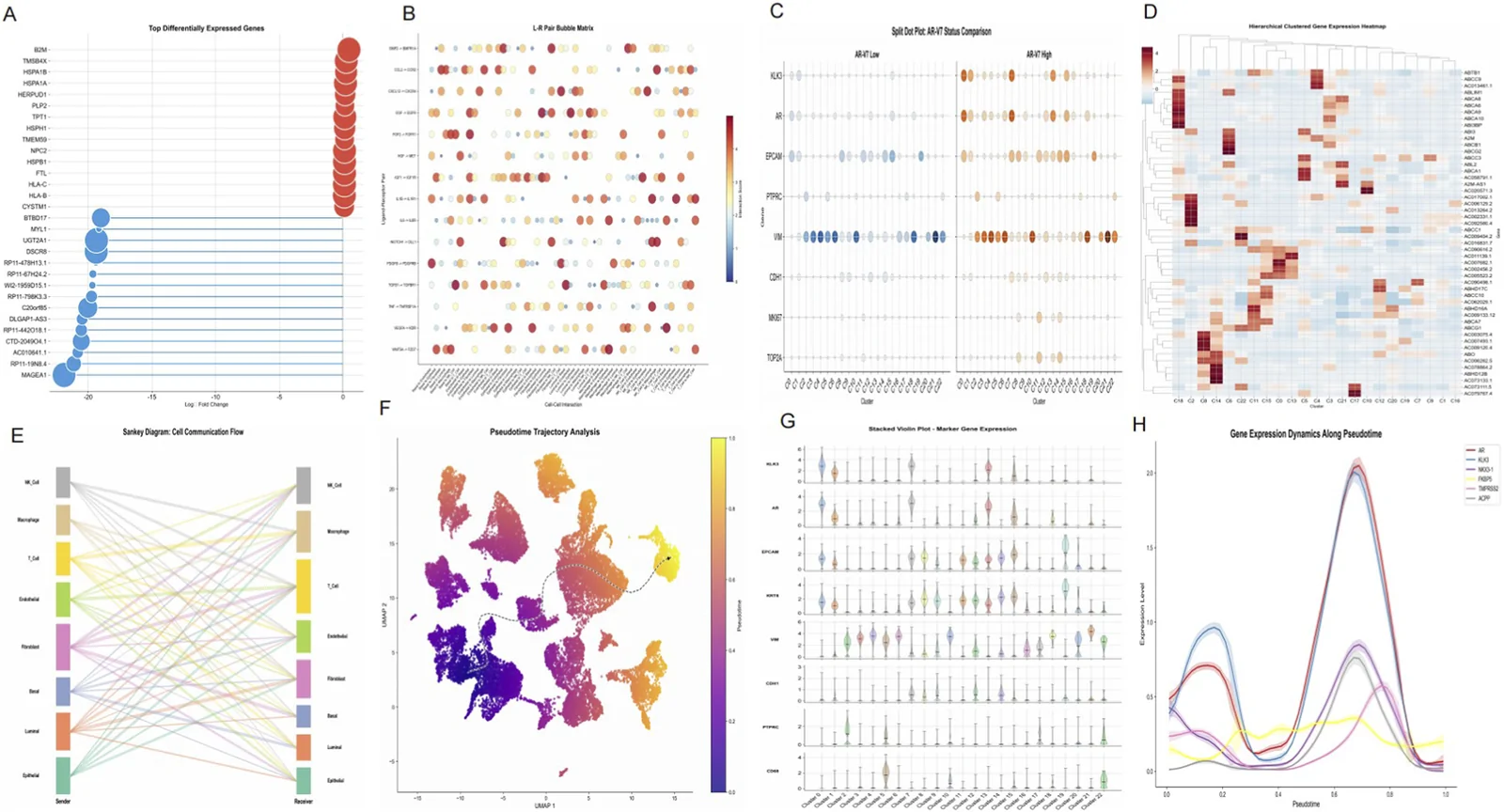
Differential expression, exploratory AR-V7-related signature visualization, pseudotime, and communication patterns. **(A)** Ranked/lollipop plot of top differentially expressed genes by log2 fold change. **(B)** Ligand–receptor pair bubble matrix across cell–cell interaction combinations. **(C)** Split dot plot comparing a computational AR-V7-related high versus low signature grouping across clusters; this grouping is transcriptomic and is not equivalent to clinically measured AR-V7 status. **(D)** Hierarchically clustered gene expression heatmap across clusters. **(E)** Sankey diagram showing inferred sender–receiver cell communication flow. **(F)** UMAP-based pseudotime trajectory with an inferred path overlaid. **(G)** Stacked violin plots of representative marker genes across clusters. **(H)** Smoothed expression dynamics of selected AR/prostate lineage genes along pseudotime.

**FIGURE 3:**
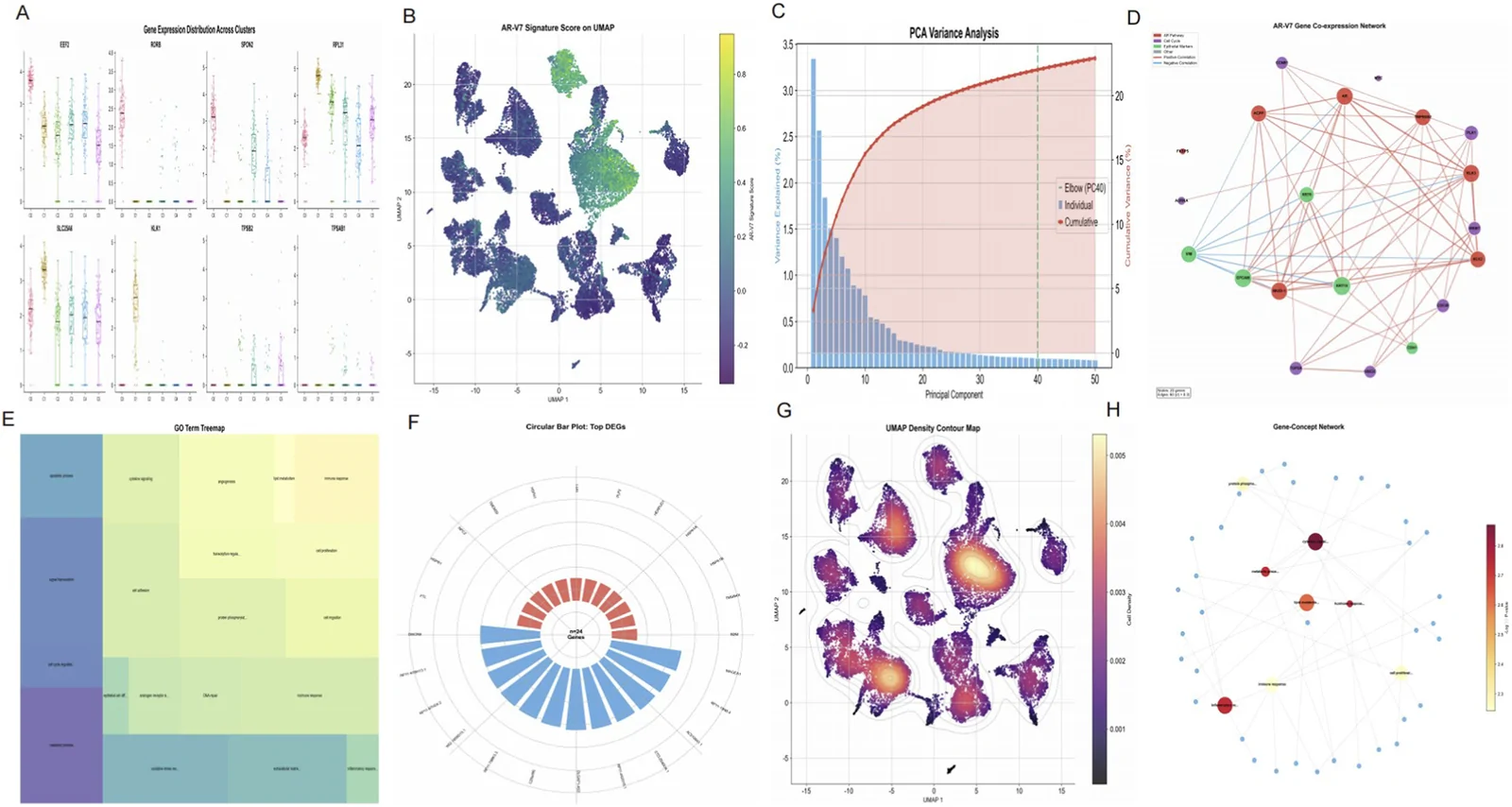
AR-associated gene expression patterns, signature mapping, enrichment, and network visualization. **(A)** Distribution of selected genes across clusters. **(B)** UMAP feature map of a computational AR-V7-related signature score; the score is exploratory and does not constitute direct AR-V7 measurement. **(C)** PCA variance analysis showing individual and cumulative explained variance and the selected component range. **(D)** AR-related gene co-expression network; edges represent positive or negative co-expression relationships rather than causal regulation. **(E)** GO term treemap summarizing enriched biological themes. **(F)** Circular bar plot of top differentially expressed genes. **(G)** UMAP density contour map. **(H)** Gene concept network linking enriched terms with associated genes.

**FIGURE 4:**
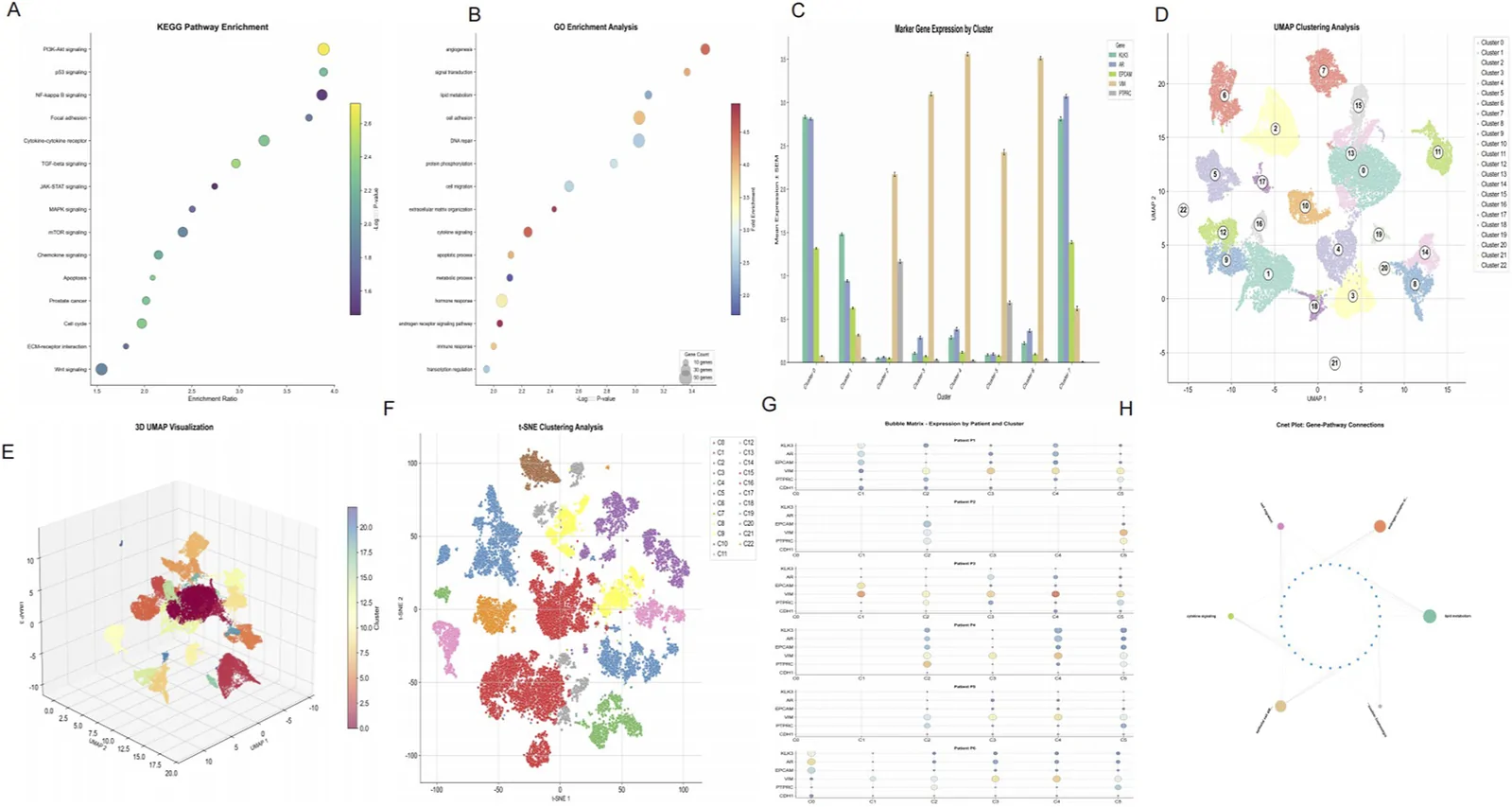
Functional enrichment, marker expression, clustering, and patient-level expression summaries. **(A)** KEGG pathway enrichment bubble plot. **(B)** GO enrichment bubble plot. **(C)** Mean expression of selected marker genes across representative clusters. **(D)** UMAP clustering of 23 transcriptional clusters (C0–C22). **(E)** Three-dimensional UMAP visualization of cluster structure. **(F)** t-SNE clustering analysis. **(G)** Bubble matrix showing selected gene expression by patient and cluster. **(H)** Gene pathway concept network connecting enriched pathways with associated genes.

### 3.3 AR-associated differential gene expression program

Differential expression visualizations identified sets of genes with large positive or negative fold changes and substantial heterogeneity across clusters (Figures 2A,D, 3F, 5F, 6D). The clustered heatmap demonstrated discrete expression modules across clusters (Figure 2D). The split dot plot and UMAP signature map display a computational AR-V7-related high/low signature and score (Figures 2C, 3B); because GSE137829 lacks validated AR-V7 measurements, these panels are interpreted only as exploratory signature-based stratification, not as clinically measured AR-V7-positive versus AR-V7-negative status. The co-expression network in Figure 3D likewise represents correlation/co-expression structure and does not demonstrate direct AR-V7 regulation.

**FIGURE 5:**
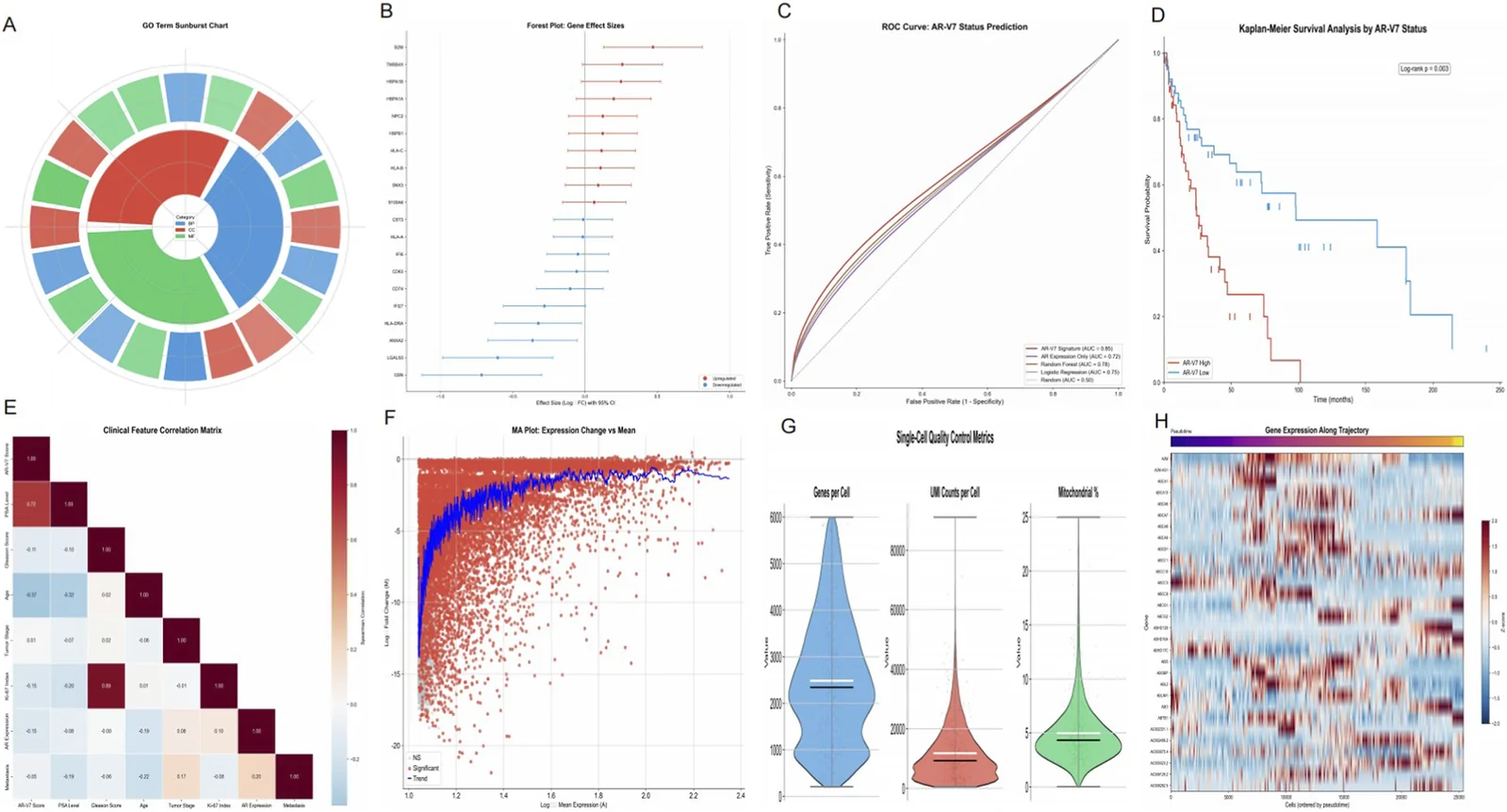
Bioinformatics-based functional, signature, quality control, and trajectory analyses. **(A)** GO term sunburst chart summarizing enriched functional categories. **(B)** Forest plot showing gene-level effect sizes with 95% confidence intervals. **(C)** ROC curves evaluating the discriminatory performance of the bioinformatics-derived AR-V7-related signature and comparator expression/signature measures. **(D)** Kaplan–Meier analysis according to computational AR-V7-related signature high versus low grouping. **(E)** Correlation matrix showing relationships among the AR-V7-related signature, PSA level, Gleason score, age, tumor stage, Ki-67 index, AR expression, and metastasis annotations. **(F)** MA plot of expression change versus mean expression. **(G)** Single-cell quality control distributions for genes per cell, UMI counts per cell, and mitochondrial percentage. **(H)** Heatmap of gene expression dynamics along pseudotime. Panels C–E are retained as exploratory bioinformatics analyses and are distinct from the deleted retrospective clinical cohort regression and composite score analyses.

**FIGURE 6:**
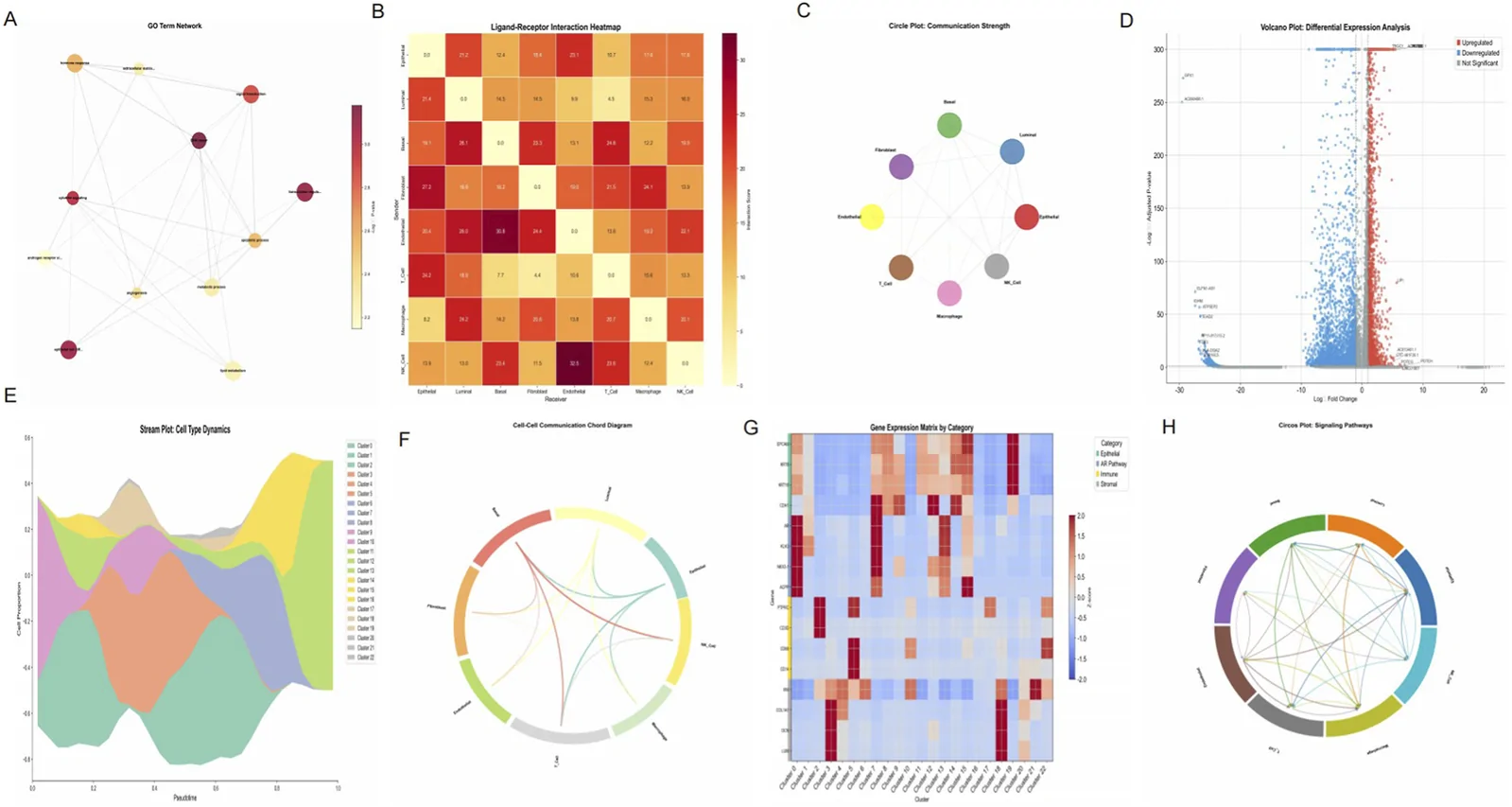
Enrichment, differential expression, cell–cell communication, trajectory, and cluster expression summaries. **(A)** GO term network. **(B)** Ligand–receptor interaction heatmap among major cell types. **(C)** Circle plot of inferred communication strength. **(D)** Volcano plot of differential expression results. **(E)** Stream plot showing changing cell state/cluster contributions along pseudotime. **(F)** Cell–cell communication chord diagram. **(G)** Cluster-level gene expression heatmap grouped by epithelial, AR pathway, immune, and stromal categories. **(H)** Circos plot summarizing connections among major signaling pathways. All communication and pathway connections are computational inferences and should be interpreted as hypothesis-generating.

### 3.4 Pseudotime trajectory and gene regulatory network analysis

Pseudotime embedding revealed a continuous trajectory spanning multiple transcriptional states (Figure 2F). Dynamic expression curves showed variation in AR pathway and prostate lineage gene expression along the inferred pseudotime axis (Figure 2H), whereas the trajectory heatmap demonstrated coordinated gene expression modules across ordered cells (Figure 5H). The stream plot further illustrated changing cluster/state composition along the inferred trajectory (Figure 6E). These analyses describe computational state transitions and should not be interpreted as longitudinal patient progression or AR-V7-specific evolution.

### 3.5 Immune-related pathway enrichment and cell–cell communication

GO and KEGG enrichment analyses highlighted pathways related to signal transduction, cell adhesion/migration, DNA repair, apoptosis, cell cycle, cytokine/chemokine signaling, PI3K–Akt, p53, NF-kappaB, TGF-beta, Janus kinase–signal transducer and activator of transcription (JAK-STAT), MAPK, mTOR, and Wnt signaling (Figures 3E-H, 4A,B-H, 5A, 6A). Ligand–receptor and cell interaction visualizations showed heterogeneous inferred communication among epithelial, luminal/basal, fibroblast, endothelial, T-cell, macrophage, and NK cell compartments (Figures 1A-H, 2B-E, 6B,C,F,H). These interaction maps are hypothesis-generating and do not establish experimentally validated PD-L1/TGF-beta/CTLA-4 signaling or AR-V7-dependent immune evasion.

### 3.6 Advanced three-dimensional visualization

Three-dimensional UMAP, two-dimensional UMAP/t-SNE, density contour, network, and gene concept visualizations provided complementary representations of cluster structure and transcriptional density (Figures 3G,H, 4D–F). These panels reinforce marked cellular state heterogeneity in CRPC. In the bioinformatics component, the AR-V7-related signature showed discriminatory performance in ROC analysis, and signature-based high/low groups showed separation in Kaplan–Meier analysis (Figures 5C,D). Correlation analysis further examined relationships between the computational signature and available annotation variables, including PSA level, Gleason score, age, tumor stage, Ki-67 index, AR expression, and metastasis (Figure 5E). These results are exploratory signature level associations and are distinct from the multivariable Cox regression, ROC/bootstrap modeling, and composite IER analyses of the deleted 78-patient retrospective clinical cohort.

### 3.7 RT-qPCR analysis of selected AR-associated gene signatures

RT-qPCR showed higher expression of KLK3, STEAP2, HOXB13, and NKX3-1 in 22Rv1 than in LNCaP cells (reported fold changes of 2.18, 2.63, 3.37, and 2.72, respectively; all *p* < 0.001) (Figure 7). These results support differences in AR-associated transcriptional programs between the two cell lines but do not isolate an AR-V7-specific effect or directly validate the immune pathways identified bioinformatically.

**FIGURE 7:**
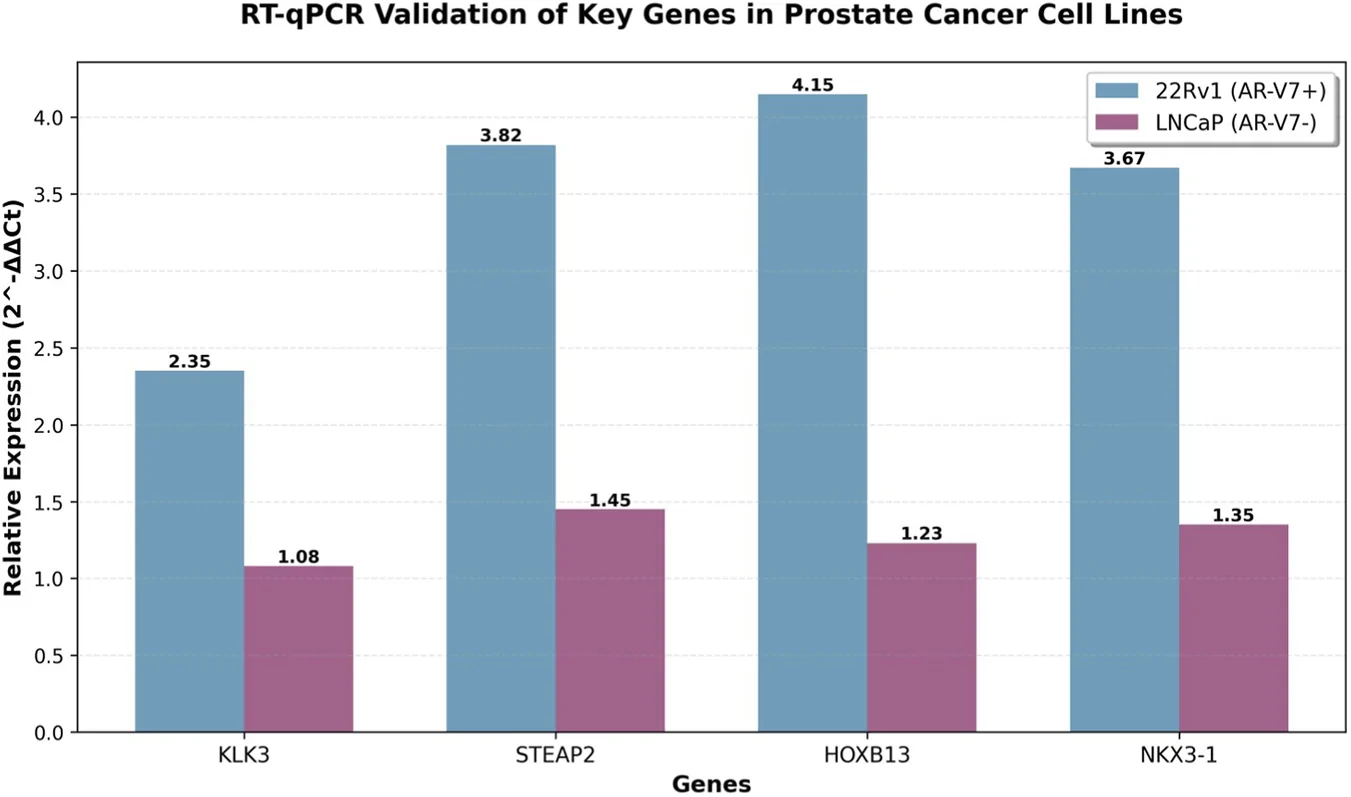
RT-qPCR analysis of selected AR-associated gene signatures. Expression of KLK3, STEAP2, HOXB13, and NKX3-1 in 22Rv1 versus LNCaP cells, normalized to GAPDH. The non-isogenic comparison is supportive and does not establish an AR-V7-specific immune mechanism.

## 4 Discussion

This revised study focuses on the molecular and bioinformatics dimensions of CRPC and preserves the single-cell analyses while removing the previously reported retrospective clinical regression study. The principal finding is that the CRPC single-cell landscape contains marked epithelial, stromal, and immune heterogeneity together with substantial variation in AR/prostate lineage transcriptional activity. The computational AR-V7-related signature further identifies a transcriptional axis that can be examined across clusters, trajectories, co-expression networks, pathway enrichment profiles, and inferred cell–cell communication. These observations support the concept that AR-V7-related biology is embedded within a broader cellular state program rather than functioning as an isolated molecular event.

The heterogeneous distribution of AR, KLK3, TMPRSS2, NKX3-1, FKBP5, KLK2, ACPP, and FOLH1 across clusters is consistent with the known diversity of AR signaling in advanced prostate cancer. Importantly, AR-V7 can emerge during castration resistance together with extensive transcriptional remodeling (Sharp et al., 2019). AR-V7-specific cistromic studies have also shown that its transcriptional output is shaped by lineage transcription factors such as HOXB13, indicating that the functional consequences of AR-V7 depend on chromatin and cellular context rather than on splice-variant abundance alone (Chen et al., 2018). This framework is relevant to the present co-expression and trajectory results, which show coordinated rather than uniform AR-related expression across CRPC states.

The differential expression and enrichment analyses extend this observation by linking AR-associated states with cell cycle, DNA damage, adhesion, migration, cytokine/chemokine, PI3K-Akt, p53, NF-kappaB, TGF-beta, JAK-STAT, MAPK, mTOR, and Wnt-related programs. These pathways are biologically plausible in treatment-resistant prostate cancer, but enrichment alone cannot establish that AR-V7 directly activates them. Prior mechanistic work has demonstrated that AR-V7 can regulate a transcriptional program distinct from full-length AR and can repress tumor-suppressive networks in CRPC (Cato et al., 2019). The current single-cell findings should therefore be viewed as a map of candidate pathway relationships that can guide targeted perturbation experiments rather than as proof of a linear AR-V7-to-immune-suppression pathway.

The immune and communication analyses are particularly relevant because advanced prostate cancer is generally characterized by a relatively immunosuppressive microenvironment. In the present dataset, inferred interactions among epithelial, T-cell, macrophage, NK cell, fibroblast, and endothelial compartments indicate that tumor cell state and immune context are tightly interconnected. Candidate TGF-beta, cytokine/JAK-STAT, chemokine, and checkpoint-related programs provide plausible routes through which resistant epithelial states could coexist with immune dysfunction. However, CellPhoneDB and related ligand–receptor methods infer communication from expression compatibility; they do not demonstrate ligand secretion, receptor engagement, downstream phosphorylation, or immune-cell functional suppression. These distinctions are essential when interpreting the communication networks.

Pseudotime analysis provides a complementary view of this heterogeneity. The continuous trajectory and dynamic expression of AR/prostate lineage genes suggest that CRPC cells occupy a spectrum of transcriptional states rather than a small number of fixed phenotypes. Such plasticity is consistent with the broader concept that treatment-resistant prostate cancer can transition between AR-dependent luminal programs and alternative lineage states. The original GSE137829 study specifically demonstrated luminal-neuroendocrine transdifferentiation at single-cell resolution (Dong et al., 2020). In the current analysis, pseudotime is therefore best interpreted as a model of state continuity within the sampled tumors, not as direct evidence that an individual patient’s tumor evolves along the displayed path.

The AR-V7-related signature analyses require similarly careful interpretation. ROC separation, Kaplan–Meier differences, and correlations with annotation variables suggest that the computational signature captures biologically meaningful variation. Nevertheless, the signature is not a direct assay of AR-V7 splice junction expression and should not be equated with validated CTC-based AR-V7 testing. This distinction is especially important because clinically validated AR-V7 assays have demonstrated treatment-specific associations in prospective and multicenter settings (Antonarakis et al., 2014; Armstrong et al., 2019), whereas the present signature was derived and evaluated within a bioinformatics framework. Its prognostic or predictive utility therefore remains exploratory until tested in an independent cohort with direct AR-V7 measurement.

The RT-qPCR findings provide limited orthogonal support for AR/prostate lineage differences between 22Rv1 and LNCaP cells. KLK3 is a canonical AR-regulated gene, whereas HOXB13 and NKX3-1 are closely related to prostate lineage identity and AR transcriptional function. AR splice variants including AR-V7 are well documented in hormone-refractory prostate cancer models (Hu et al., 2009). However, 22Rv1 and LNCaP are genetically distinct cell lines, so higher KLK3, STEAP2, HOXB13, or NKX3-1 expression cannot be attributed specifically to AR-V7. Moreover, these genes do not directly validate PD-L1, TGF-beta, IL-6/JAK-STAT, CTLA-4, or interferon/CXCL signaling. The RT-qPCR section is therefore appropriately positioned as supportive molecular context rather than mechanistic validation.

The translational relevance of these findings lies in hypothesis generation. PSMA-targeted radioligand therapy can induce tumor cell damage and may create opportunities for immune modulation, whereas early clinical work has explored combination strategies such as 177Lu-PSMA-617 followed by pembrolizumab (Aggarwal et al., 2023). The present data do not demonstrate that an AR-V7-related signature predicts response to radioligand therapy, because the retrospective RLT outcome cohort has been removed. Instead, the transcriptomic results identify candidate tumor-state and immune interaction features that could be prospectively tested in future studies combining direct AR-V7 measurement, PSMA imaging, treatment response, and longitudinal immune profiling.

Several limitations should be emphasized. First, GSE137829 contains only six CRPC patients, and single-cell analyses can generate large numbers of cells without increasing the number of independent patients. Second, direct AR-V7 status is not provided in the public metadata, making the AR-V7-related score a computational proxy rather than a validated molecular assay. Third, pseudotime, co-expression, enrichment, and ligand–receptor analyses are inferential and susceptible to model assumptions. Fourth, the bioinformatics ROC, survival, and correlation analyses require independent validation and should not be interpreted as clinical model development. Fifth, the RT-qPCR comparison is non-isogenic and lacks AR-V7 knockdown, overexpression, CRISPR editing, cytokine secretion assays, PD-L1 protein measurement, or immune-cell co-culture. Finally, external single-cell validation in an independent AR-V7-annotated CRPC cohort is not available.

Future work should therefore prioritize prospectively collected CRPC specimens with direct AR-V7 splice junction quantification, paired scRNA-seq or single-cell multi-omic profiling, and standardized clinical annotation. Isogenic AR-V7 perturbation models will be required to determine whether the candidate immune pathways identified here are downstream consequences of AR-V7 itself. Functional experiments should measure checkpoint proteins, cytokine secretion, antigen-presentation machinery, T-cell activation, and macrophage polarization. Such studies would allow the present computational associations to be converted into testable mechanistic models and could establish whether AR-V7-related tumor states have value for therapeutic stratification. Future trajectory analyses should also incorporate sensitivity analyses across alternative inference frameworks, as method choice can materially affect reconstructed trajectories (Saelens et al., 2019).

Taken together, the revised analysis supports a model in which AR/AR-V7-related transcriptional variation is intertwined with CRPC cellular plasticity and the surrounding immune/stromal ecosystem. The strength of the study lies in the breadth of complementary single-cell analyses; its principal constraint is the absence of direct AR-V7 annotation and functional perturbation. Maintaining this distinction between computational association and biological causation provides a more rigorous basis for future validation.

## 5 Conclusion

The revised analysis identifies AR-associated transcriptional and cellular features in CRPC but does not establish that AR-V7 causally orchestrates immune suppression. Direct AR-V7 perturbation experiments and prospectively annotated AR-V7-defined patient datasets are required before AR-V7-specific mechanistic or clinical conclusions can be supported.

## Data Availability

The publicly available single-cell RNA-sequencing dataset analyzed in this study is available in the NCBI Gene Expression Omnibus under accession number GSE137829.
